# A Pilot Study of Hypofractionated Radiosurgery for Trigeminal Neuralgia

**DOI:** 10.7759/cureus.53061

**Published:** 2024-01-27

**Authors:** Sophia N Shah, Praneet Kaki, Sohan S Shah, Sunjay A Shah

**Affiliations:** 1 Radiation Oncology, Christiana Care Health System, Newark, USA

**Keywords:** stereotactic radiosurgery (cyberknife®), bni pain intensity, hypoesthesia, hypofractionated stereotactic radiosurgery, trigeminal neuralgia

## Abstract

The primary late toxicity of radiosurgery treatment for trigeminal neuralgia (TN) is facial numbness due to trigeminal nerve dysfunction. Although most patients prefer loss of facial sensation to TN, severe loss of facial sensation can be debilitating. In order to try to obtain high pain control rates while minimizing the risk of late facial numbness, we elected to treat patients on the distal trigeminal nerve with a three-fraction regimen over consecutive days instead of one fraction. Our goal was to relieve the pain while also allowing the trigeminal nerve time to repair radiation damage between treatments in an attempt to minimize the risk of permanent facial numbness. Patients in a pilot study, approved by an Institutional Review Board (IRB), received a treatment regimen of 99 Gy, administered in three consecutive daily fractions of 33 Gy each, with the dosage targeted to the 80% line. This dose was selected to approximate a biologically equivalent dose of 80 Gy maximal dose to the trigeminal nerve. Forty-eight patients were treated with CyberKnife Radiosurgery (CKRS; 99 Gy/3 fractions) for TN from 2016 to 2022, with at least one year of follow-up. The Barrow Neurological Institute (BNI) scale was used to assess facial pain, and Kaplan-Meier analysis was used to assess adequate pain relief. Thirty-eight (84%) patients experienced adequate pain relief, defined as a BNI score of I-IIIb, after a median of 1.5 months following CKRS. Treatment failure (BNI=IV-V) occurred in 12 (25%) patients after a median of 6 months following initial pain relief. The actuarial probability of pain relief at 6, 12, and 24 months post-CKRS were 87.4%, 83.7%, and 83.7%, respectively. Facial numbness was experienced in 24 (50%) cases after a median of 10 months following CKRS. Typical facial pain (p=0.034) and vascular compression (p=0.039) were the only predictors of better treatment outcomes using univariate Cox survival analysis, and vascular compression (p= 0.037) was the only predictor in multivariate Cox survival analysis. Hypofractionated treatment to the distal trigeminal nerve segment does not appear to offer an advantage in treating TN, due to similar rates of pain relief but with an unacceptably high rate of late facial numbness.

## Introduction

Trigeminal neuralgia (TN) is a chronic, neuropathic disease characterized by recurrent, lancinating, unilateral facial pain that can last from a few seconds to minutes [1]. With an annual incidence of 10,000-15,000 patients in the United States, it affects women more than men [2]. The first line of treatment includes antiepileptic medications like carbamazepine, gabapentin, and oxcarbazepine [3]. For patients who fail medical management or are intolerant to medication, more invasive therapies are available [4]. The gold standard treatment, microvascular decompression (MVD), relieves the impingement of a blood vessel on the trigeminal nerve and boasts a 90% success rate [5]. Age, however, is a significant risk factor; patients under 65 have a mortality rate of 0.13%, whereas it rises to 1.16% for those over 75 [6]. Another treatment option for TN is percutaneous rhizotomy, which damages the pain-transmitting nerve fibers, using techniques like radiofrequency thermocoagulation, balloon compression, and glycerol rhizotomy, with success rates between 75 to 90% [5].

Stereotactic radiosurgery (SRS) is a non-invasive treatment suitable for both malignant and benign conditions and involves focusing multiple high-energy radiation beams onto the treatment area [7]. SRS is typically recommended for TN patients who are not ideal surgical candidates, such as the elderly or those without clear vascular compression on MRI, and for those who have not responded to previous treatments like microvascular decompression or rhizotomy. Instruments used in SRS include the Gamma Knife, a linear accelerator, and the CyberKnife device. The Gamma Knife involves placing a helmet with screws onto the patient's head to immobilize it, ensuring the radiation converges at a single isocenter to minimize damage to surrounding tissue [8]. Traditionally, this has been done in one fraction due to the invasive immobilization. On the other hand, with the CyberKnife linear accelerator device, the patient undergoes real-time tracking using stereoscopic imaging. Using non-isocentric planning, it is relatively easy to treat a target with complex morphology. The device readily permits multi-fraction treatment since there is no need for an invasive head frame [9]. 

The primary late toxicity of SRS treatment for TN is late facial numbness due to trigeminal nerve damage. Although most patients prefer loss of facial sensation to TN, severe loss of facial sensation can be debilitating due to the risk of corneal abrasions, dysgeusia, and recurrent bleeding from biting the tongue or oral mucosa. In an effort to maintain high pain control rates while minimizing the risk of late facial numbness, we elected to treat patients on the distal trigeminal nerve with a three-fraction regimen over consecutive days instead of one fraction. Fractionation is a standard principle of radiation oncology used to maintain acute effects while decreasing the risk of late effects [10]. Our goal was to relieve acute pain symptoms while allowing the trigeminal nerve time to repair radiation damage between treatments, thereby minimizing the risk of late facial numbness. Patients were treated in an IRB-approved pilot study with a dose of 99 Gy prescribed to the 80% line in three consecutive daily fractions of 33 Gy each. This dose was chosen to approximate a biologically equivalent maximum dose of 80 Gy to the trigeminal nerve [11].

## Materials and methods

Patient selection 

Since 2008, TN patients have been treated at our institution with the CyberKnife robotic radiosurgery device. Patients who were treated with a dose of 99 Gy in three fractions and had a follow-up of at least one year were selected for inclusion in this study. Patients were followed up in person or by phone in accordance with this Christiana Care Health Services IRB-approved retrospective study.

CyberKnife radiosurgery (CKRS) treatment

**Figure 1:**
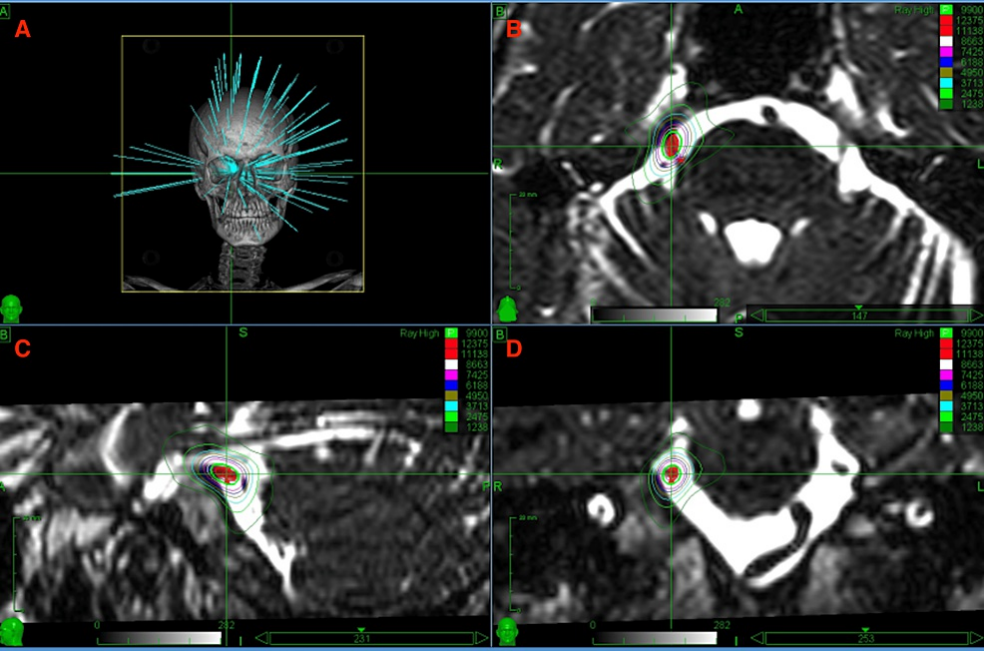
(A) Example of a CyberKnife radiation plan. (B) Radiation plan in the axial plane. (C) Radiation plan in the sagittal plane. (D) Radiation plan in the coronal plane.

**Figure 2:**
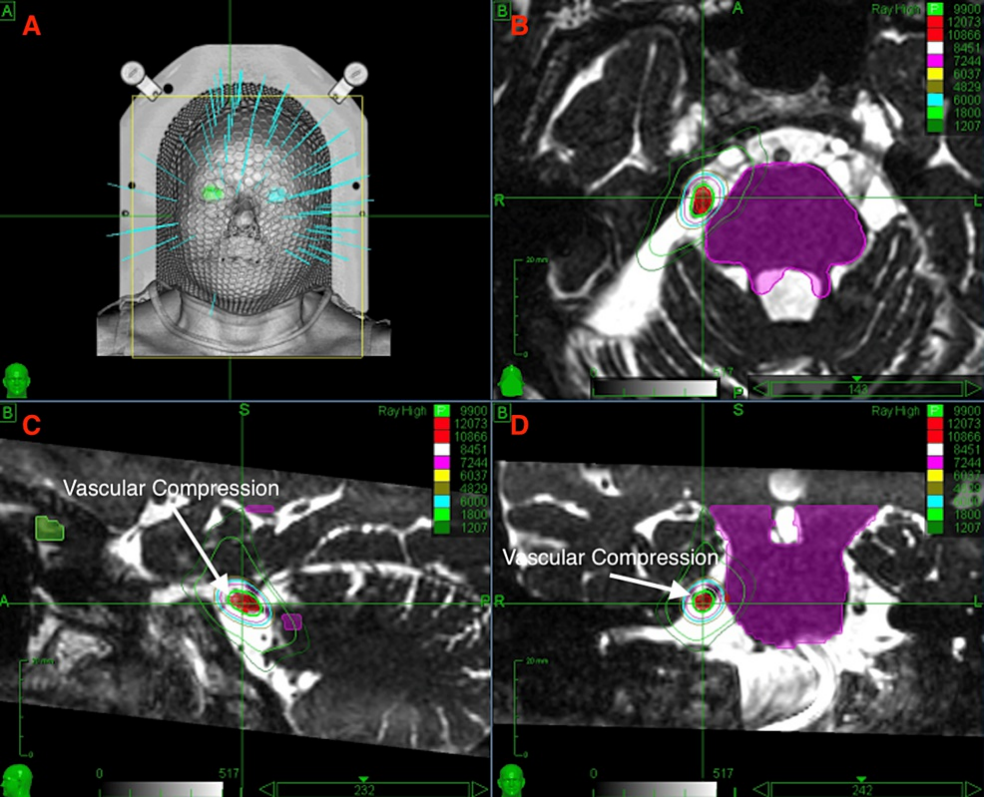
(A) Example CyberKnife radiation plan. (B) Radiation plan in the axial plane. (C) Radiation plan in the sagittal plane. (D) Radiation plan in the coronal plane.

Treatment was performed using a CyberKnife device (Accuray Inc., Sunnyvale, California, United States). Before treatment, planning for each intervention included a high-resolution (1-mm slice thickness) CT scan and 3D constructive interference in steady state (CISS) T2-weighted images on a Siemens MRI scanner. For patients in whom MRI was not possible, a CT myelogram was performed for planning. The retro-gasserian portion of the trigeminal nerve was targeted for treatment. The treatment was then planned on MultiPlan or Precision software (Accuray Inc.) with both CT and 3D CISS MRI (Figure 1). CKRS dose planning was conducted by a radiation oncologist, a neurosurgeon, and a medical physicist. The determination of vascular compression of the nerve was made by the radiation oncologist after reviewing the 3D CISS data set (Figure 2). The average maximum CKRS dose administered (Dmax) was 122.39 Gy (SD = 5.37) in three consecutive daily fractions. All treatment was completed within one week.

Outcome measures 

The Barrow Neurological Institute (BNI) scale was used to assess patient-reported facial pain from I to V: I, no trigeminal pain, no medications; II, occasional facial pain, not requiring medication; IIIa, no pain, continued medication; IIIb, persistent pain, controlled with medications; IV, some pain, not adequately controlled with medication; and V, severe pain/no pain relief. Treatment failure due to pain recurrence was defined as a BNI score of IV or V; success/pain relief was characterized by a score of I-IIIb (Table 1). The BNI scale was also used to measure facial numbness from I to IV: I, no facial numbness; II, some facial numbness, not bothersome; III, some facial numbness, somewhat bothersome; IV, facial numbness, very bothersome. A complication due to facial numbness was defined as a BNI score of III or IV (Table 2) [12].

**Table 1:** Barrow Neurological Institute (BNI) pain intensity scores.

BNI Pain Intensity Scale	
Score	Pain Description
I	No pain, no medications
II	Occasional Pain, no medications required
III	Some pain, adequately controlled with medications
IIIa	No pain, continued medication
IIIb	Persistent pain, controlled with medication
IV	Some pain, not adequately controlled with medications
V	Severe pain or no pain relief

**Table 2:** Barrow Neurological Institute (BNI) numbness scale.

BNI Numbness Scale	
Score	Numbness Description
I	No facial numbness
II	Mild facial numbness that is not bothersome
III	Somewhat bothersome facial numbness
IV	Very bothersome facial numbness

Statistical analysis 

Kaplan-Meier survival analysis was used to delineate the extent of adequate pain relief following CKRS. Data from the patient's most recent follow-up were used to calculate their respective time intervals. Patients were censored if they did not report a BNI pain score of IV or V at this follow-up. Log-rank tests were used to compare long-term pain relief outcomes among patients with atypical versus typical pain and those with and without vascular compression. Univariate and multivariate Cox regression analyses were performed to identify predictors of treatment success or failure. Statistical significance was defined as a p-value less than 0.05. R software was used to perform all statistical analyses.

## Results

Case characteristics

Demographics and essential case characteristics are outlined in Table 3. There are 48 total cases, of which 31 (65%) are female and 17 (35%) are male. The average age at CKRS administration was 67.5 years. TN pain was experienced on the left side in 20 (42%) cases and on the right side in 28 (58%) cases. There was a simple division of TN pain in 20 (49%) patients, compared to a multiple division in 21 (51%) patients. Vascular compression was identified in 33 (72%) cases, determined by the radiation oncologist’s interpretation of clear compression of the nerve root by the blood vessel. Most (90%) of the patients had an idiopathic etiology of TN, as only 5 (10%) of the cases also had multiple sclerosis (MS). Eighteen (37.5%) of the patients had prior surgery for their diagnosis. Prior microvascular decompression (MVD), percutaneous rhizotomy (PR), and MVD + PR were all evenly reported, with six (12%) patients undergoing each.

**Table 3:** Demographic and TN characteristics. ^1^Values indicate the number of patients (%) unless otherwise indicated. MS: Multiple sclerosis; MVD: Microvascular decompression; PR: Prior Rhizotomy; CKRS: CyberKnife Radiosurgery; TN: Trigeminal neuralgia.

Characteristics	Value^1^
Age in years (SD)	67.48 (14.10)
Sex	
Male	17 (35%)
Female	31 (65%)
Laterality	
Left	20 (42%)
Right	28 (58%)
TN Division	
Multiple	21 (51%)
Simple	20 (49%)
Vascular Compression	33 (72%)
Etiology	
Idiopathic	43 (90%)
MS	5 (10%)
Prior Surgery	
MVD	6 (12%)
PR	6 (12%)
MVD and PR	6 (12%)
None	30 (62%)
Average CKRS Dose in Gy (SD)	122.39 (5.37)

Follow-up and pain relief following CKRS 

The median time to the most recent follow-up was 22 months (range 1-77 months). Of the 45 patients with sufficient data, 38 (84%) experienced initial adequate pain relief after a median period of 1.5 months following CKRS treatment. Among these patients, four had an MS-related etiology, and 34 were idiopathic. There were 12 patients (25%) who experienced treatment failures, defined as BNI scores of IV or V, after a median period of six months following initial pain relief (Table 4).

**Table 4:** Clinical outcomes following CKRS. ^1^Values indicate the number of patients (%) unless otherwise indicated. ^2^Wilcoxon rank sum test; Fisher's exact test. MS: Multiple sclerosis; CKRS: CyberKnife Radiosurgery.

Characteristic	N^1^	Overall, N = 48^1^	Idiopathic, N= 43^1 ^	MS, N=5^1^	P-value^2^
Median follow-up in months (IQR)	48	22 (10, 47)	22 (10, 47)	16 (9, 31)	0.4
Initial adequate pain relief	45	38 (84%)	34 (85%)	4 (80%)	>0.9
Median time from CKRS to pain relief in months (IQR)	39	1.50 (0.75, 2.00)	1.00 (0.75, 2.00)	1.63 (1.19, 3.56)	0.6
Experienced CKRS treatment failure	48	12 (25%)	11 (26%)	1 (20%)	>0.9
Median time from adequate initial pain relief to failure of CKRS in months (IQR)	5	6.00 (5.75, 10.50)	6.00 (5.75, 10.50)	NA (NA, NA)	
Experienced facial numbness following CKRS	48	24 (50%)	20 (47%)	4 (80%)	0.3
Median time from CKRS to onset of facial numbness in months (IQR)	24	10 (6, 15)	8 (6, 12)	17 (10, 26)	0.2

Maintenance of adequate pain relief after CKRS 

**Figure 3:**
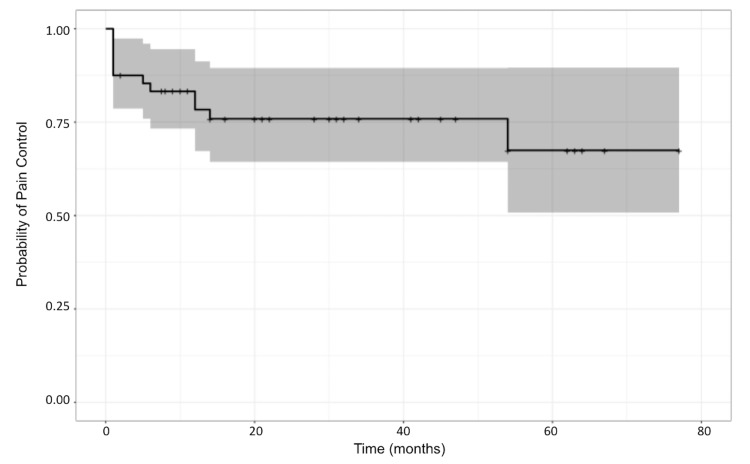
Actuarial pain control rates of patients after treatment.

**Figure 4:**
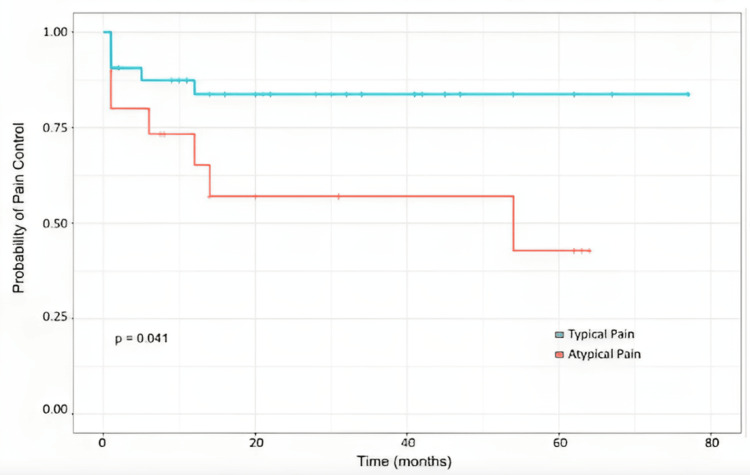
Actuarial pain control rates of patients after treatment by pain type.

**Figure 5:**
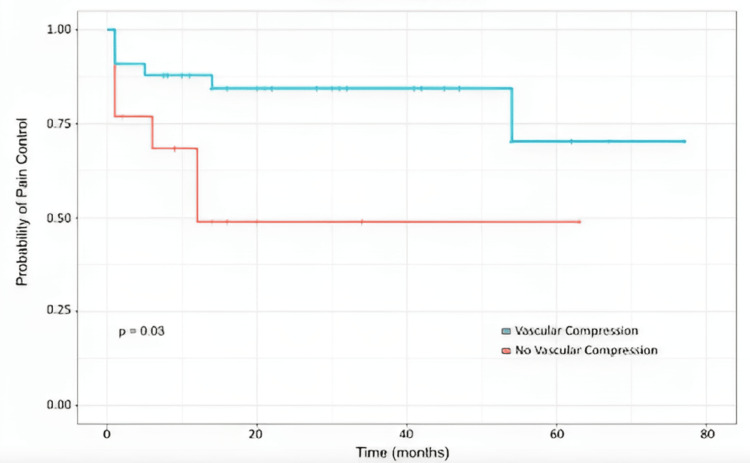
Actuarial pain control rates of patients after treatment by vascular compression.

The Kaplan-Meier survival curve for the whole cohort is depicted in Figure 3. The median duration of adequate pain control following CKRS is 25 months. The actuarial probabilities of pain control at 6 months, 12 months, and 24 months were 83.2%, 78.3%, and 75.9%, respectively. Stratified Kaplan-Meier curves were also created to compare outcomes for patients experiencing typical versus atypical pain (Figure 4). These curves showed that patients with typical pain demonstrate better long-term pain relief than those with atypical pain (p = 0.024). The actuarial probabilities for pain relief for cases with typical pain at 6 months, 12 months, and 24 months are 87.4%, 83.7%, and 83.7%, respectively. All the patients who had follow-up visits after 12 months were censored, thereby not demonstrating further pain recurrence. For atypical pain cases, the actuarial probabilities at the same time intervals are 71.4%, 62.5%, and 53.6%, respectively. A stratified survival curve was created to compare patients with and without vascular compression. Patients with vascular compression were found to have more favorable long-term pain management outcomes compared to cases without vascular compression (p = 0.03). The actuarial probabilities at 6, 12, and 24 months for cases with vascular compression were 87.9%, 87.9%, and 84.4%, respectively, compared to 68.4%, 48.8%, and 48.8% for those without vascular compression (Figure 5).

Predictors of CKRS success/failure

Cox survival analysis was used to identify predictors of CKRS treatment outcomes, with the results presented in Table 5. Univariate analysis identified two predictors for better treatment outcomes: the presence of vascular compression (p = 0.039) and a typical presentation of pain (p = 0.034). However, on multivariate analysis, only vascular compression was associated with favorable treatment outcomes. 

**Table 5:** Univariate and multivariate analyses of outcome for patients at the final follow-up. MS: Multiple sclerosis; MVD: Microvascular decompression.

	Univariate			Multivariate		
Predictive Factor	HR	95% CI	P-value	HR	95% CI	P-value
Age, years	0.97	0.93-1.001	0.057	0.94	0.89-1.01	0.075
Is Male	2.4	0.76-7.6	0.14	0.26	0.031-2.22	0.22
Right-Sided Laterality	1.49	0.45-4.95	0.52	0.65	0.099-4.23	0.65
Simple TN Division	1.85	0.54-6.31	0.33	0.82	0.17-4.00	0.8
MS Etiology	0.92	0.12-7.22	0.94	0.43	0.031-5.93	0.53
Prior MVD	1.93	0.6-6.15	0.27	6.39	0.82-49.9	0.077
Prior Rhizotomy	0.91	0.25-3.37	0.89	0.35	0.53-2.33	0.28
Vascular Compression	0.3	0.10-0.94	0.039	0.1	0.012-0.87	0.037
Typical Pain	0.29	0.09-0.91	0.034	0.96	0.14-6.60	0.96
Total Gy Dose	1.07	0.95-1.20	0.26	0.97	0.84-1.12	0.64
Brainstem Max	1	0.99-1.001	0.225	1	1.00-1.00	0.15
Nerve Length Treated, mm	1.05	0.78-1.40	0.77	0.84	0.44-1.59	0.59

Late complications after CKRS

Twenty-four (50%) of the cases experienced new facial numbness or exacerbated pre-existing numbness following CKRS treatment, defined as BNI scores of IV or V. The median time from CKRS treatment to the onset of facial numbness was 10 months (range 3-47 months). No other cranial neuropathies or late toxicities were reported.

## Discussion

Radiosurgery is an alternate treatment option to surgery in high-risk individuals and is considered the least invasive option for medically intractable TN. A study from the University of Pittsburgh is representative of multiple published retrospective series describing treating TN with Gamma Knife radiosurgery. In their report, one fraction of either 70 or 90 Gy was administered to a single 4-millimeter isocenter located at the dorsal root entry zone. 68 patients were treated, and 27 patients received a dose of 70 Gy while 41 patients received a dose of 90 Gy. The length of follow-up ranged from 2 to 36 months with a mean of 14.4 months. Of the high-dose patients, 61% remained pain-free whereas 41% of low-dose patients remained pain-free suggesting greater pain relief is associated with a higher dose. In addition, 44% of low-dose patients underwent additional post-radiation treatments for their TN. However, 32% of the high-dose patients experienced facial numbness compared to only 3.7% of the low-dose patients [13]. This radiosurgery technique is the most commonly used method for treatment of TN. 

There is no consensus on the area of the trigeminal nerve that should be targeted [14]. Dr. Adler from Stanford described their experience with the CyberKnife device using an alternate technique of treating a distal segment of the trigeminal nerve (retro-gasserian technique) with nonisocentric planning instead of a single isocenter. After a mean follow-up period of 14.7 months, patient-reported outcomes were excellent in 33 patients (72%) [15]. Two studies, one by Karam SD et al. at Georgetown University Hospital [16] and one by Romanelli P et al. in Milan [17], replicated the Stanford technique, and both results indicated the treatment’s long-term effectiveness at treating pain but with relatively high subsequent rates of facial numbness, 28% and 20.1%, respectively, after radiosurgery.

Our pilot study was an attempt to maintain the same early responding pain benefit while utilizing hypofractionation to attempt to minimize the late onset of facial numbness due to microvascular fibrosis. We chose to pilot a three-fraction regimen. In Figure 9, our results are compared to the largest published CKRS studies on TN treatment, by Romanelli P et al. [17], Conti A et al. [18], and Guillemette A et al. [11]. The mean follow-up periods for the studies were 26, 38, and 36.7 months, respectively, while our study had a median follow-up period of 22 months. A total of 84% of our patients reported initial adequate pain relief at an average of 1.5 months following treatment. Romanelli P et al. [17], Conti A et al. [18], and Guillemette A et al. [11] studies reported pain relief rates of 92%, 88.2%, and 86.9% respectively. Only the Guillemette A et al. study [11] provided a median time to pain relief of five weeks. When looking at only patients with idiopathic TN, 90% of our study’s patients had initial adequate pain relief at a median of 1.25 months post-treatment. Again, the Guillemette A et al. study [11] was the only study to differentiate between different etiologies of TN; 89.4% of idiopathic TN patients had initial adequate pain relief at a median of 1.5 months. Our rates of initial pain control are consistent with the literature.

The actuarial probabilities of pain control for this study at 6 months, 12 months, and 24 months were 83.2%, 78.3%, and 75.9%, respectively. This is slightly inferior to the results from the Romanelli A et al. [17] and Conti A et al. [18] studies. Romanelli A et al. [17] reported 6-month, 12-month, and 24-month actuarial pain control of 92%, 87%, and 82%, respectively while Conti A et al. [18] reported a 6-month, 12-month, and 24-month actuarial pain control of 96.8%, 90.9%, and 84.2%. However, the Guillemette A et al. [11] study reported a 12-month actuarial pain control rate of 77%, similar to our findings. 

On multivariate analysis, we found that vascular compression was the only predictor of more favorable long-term outcomes following treatment (p = 0.03). This is consistent with the data from the surgical series. Presumably, this is because some of the cases without vascular impingement are caused by central ideology not amendable to the treatment of the trigeminal nerve. The actuarial probability in this study for maintenance of pain control at 6, 12, and 24 months for cases with vascular compression were 87.9%, 87.9%, and 84.4%, respectively, compared to 68.4%, 48.8%, and 48.8% for those without vascular compression. The determination of vascular compression of the nerve was made by the radiation oncologist after a review of the 3D CISS data set. In our experience, there were several cases of trigeminal nerve vascular conflict that were not mentioned in the official radiology reading but were clearly seen in a review of the 3D high-resolution data set in multiple dimensions. This likely accounted for the higher rate of vascular compression seen in our study and the strong association with pain relief benefits. 

The primary complication from treatment in our study was unilateral facial numbness. In this study, 50% of patients suffered from numbness (BNI III, IV) at a median of 10 months after the onset of treatment. The studies by Romanelli P et al. [17], Conti A et al. [18], and Guillemette A et al. [11] reported only 6.1%, 18%, and 13.7% of patients experiencing facial numbness, respectively. However, the Romanelli P et al. [17] and Conti A et al. [18] studies did not distinguish between different BNI numbness grades, while the Guillemette A et al. study [11] showed data for each number on the BNI scale. There is considerable subjectivity in the scoring of facial numbness and well-documented differences in scoring between physicians and patients. Our facial numbness scoring was obtained largely by careful telephone follow-up of patients, which may have resulted in higher scoring than other reported studies. Nevertheless, the late risk of facial numbness was considerably higher than reported in other studies and may be due to an entire segment of the distal trigeminal nerve being treated to a high biologically equivalent dose. This suggests no benefit from a three-fraction hypofractionation regimen at this dose level in terms of preventing late facial numbness (Table 6).

**Table 6:** Literature review of retrospective studies evaluating stereotactic radiosurgery. ^1^Only mean follow-up period in months was reported.

Study	Median follow-up period in months	Median D_max_ in Gy	Number of Treatment Fractions	Number of Patients Treated	Pain Relief at 6 months (%)	Pain Relief at 12 months (%)	Hypoesthesia (%)
Romanelli P et al., (2019) [17]	14.7^1^	71.3	1	496	92	87	20.1
Conti A et al., (2020) [18]	38	72.4	1	262	96.8	90.9	18
Guillemette A et al., (2022) [11]	36.7	80	1	166	NA	77	13.7
Shah SN et al., (2023) (current study)	22	122.39	3	48	83.2	78.3	50

Limitations 

The inherent retrospective design of this study imposes limitations, namely recall bias, as pain-related data were self-reported by patients during long-term follow-up visits. Longer follow-up periods are also necessary to establish a well-founded pattern in CKRS outcomes over several years; this is tied to the intrinsic limitations brought on by the relatively low sample size of the cohort included in this study.

Future studies 

This study serves as a small-sample retrospective analysis on the outcomes of TN patients following CKRS. However, its scope is limited, and more research on this population is certainly necessary. A prospective study, in which patients are administered standardized questionnaires at consistent time intervals, can better depict and characterize the long-term outcomes of this treatment. Moreover, complications in addition to hypoesthesia could be carefully tracked to expand the scope of such a study. It may also be relevant to evaluate the outcomes based on the length of the nerve treated with CKRS.

## Conclusions

There does not appear to be any benefit from hypofractionation to the distal trigeminal nerve in terms of reducing the risk of late facial numbness after CKRS for medically refractory TN. While the pain relief rates appear similar, there is a considerably higher risk of late facial numbness upon long-term follow-up. The presence of vascular compression and typical pain were associated with more favorable long-term pain control. Despite theoretical advantages in terms of reducing the risk of late effects, we cannot recommend a hypofractionated technique at this time. A pilot study of hypofractionation with dose de-escalation is currently in progress.
